# The influence of childhood asthma on adult height: evidence from the UK Biobank

**DOI:** 10.1186/s12916-022-02289-1

**Published:** 2022-03-22

**Authors:** Wenwen Chen, Huazhen Yang, Can Hou, Yajing Sun, Yanan Shang, Yu Zeng, Yao Hu, Yuanyuan Qu, Jianwei Zhu, Fang Fang, Donghao Lu, Huan Song

**Affiliations:** 1 Division of Nephrology, Kidney Research Institute, State Key Laboratory of Biotherapy and Cancer Center, West China Hospital, Sichuan University, Chengdu, 610041 China; 2 West China Biomedical Big Data Center, West China Hospital, Sichuan University, Guo Xue Lane 37, Chengdu, 610041 China; 3 Med-X Center for Informatics, Sichuan University, Chengdu, 610041 China; 4 Department of Orthopedic Surgery, West China Hospital, Sichuan University, Chengdu, 610041 China; 5 Institute of Environmental Medicine, Karolinska Institute, 17177 Stockholm, Sweden; 6 Department of Epidemiology, Harvard T H Chan School of Public Health, Boston, MA 02215 USA; 7 Center of Public Health Sciences, Faculty of Medicine, University of Iceland, 101 Reykjavík, Iceland

**Keywords:** Childhood asthma, Adult height, Genetic heterogeneity

## Abstract

**Background:**

To elucidate the influence of childhood asthma on adult height after consideration of genetic heterogeneity in height.

**Methods:**

Based on the UK Biobank, we conducted a matched cohort study, including 13,602 European individuals with asthma diagnosed before 18 years old and 136,008 matched unexposed individuals without such an experience. Ascertainment of asthma was based on self-reported data (97.6%) or clinical diagnosis in healthcare registers (2.4%). We studied three height outcomes, including (1) the attained adult height (in centimeters), (2) the height deviation measured as the difference between a person’s rank of genetically determined height (based on generated polygenetic risk score) and their rank of attained adult height in the study population (deviation in % of height order after standardization), and (3) the presence of height deficit comparing genetically determined and attained height (yes or no). We applied linear mixed-effect models to assess the associations of asthma diagnosed at different ages with attained adult height and height deviation, and conditional logistic regression models to estimate the associations of asthma with the risk of height deficit.

**Results:**

40.07% (59,944/149,610) of the study participants were born before 1950, and most of them were men (57.65%). After controlling for multiple covariates, childhood asthma was associated with shorter attained adult height, irrespective of age at asthma diagnosis. However, in the analysis of height deviation (deviation in %), we observed the greatest height deviation among individuals with asthma diagnosed before 4 years of age (− 2.57 [95% CI − 4.14 to − 1.00] and − 2.80 [95% CI − 4.06 to − 1.54] for the age of ≤ 2 and 3–4 years, respectively). The magnitude of height deviation in relation to asthma declined thereafter and became null after age 6. Similarly, there was a statistically significant height deficit in relation to an asthma diagnosis at ages ≤ 2 and 3–4 (odds ratios = 1.21, 95% CI 1.04 to 1.40, and 1.15, 95% CI 1.02 to 1.29) but not thereafter. The result pattern was similar when separately analyzing asthma with or without inhaled glucocorticoid (ICS) use, despite that the estimates were consistently stronger among asthma individuals who used ICS.

**Conclusions:**

Our results suggest a notable association of childhood asthma, primarily asthma diagnosed at an early age, with adult height, after consideration of genetic heterogeneity in height and use of ICS. This finding highlights the need for surveillance on the growth problems among children with asthma.

**Supplementary Information:**

The online version contains supplementary material available at 10.1186/s12916-022-02289-1.

## Background

Characterized by airway hyper-reactivity and inflammation, as well as reversible airflow obstruction, asthma was found to affect more than 272 million people worldwide in 2017, with a particularly high prevalence among children (~ 7.0%) [1, 2]. Given the large population of children with asthma, there have been long-lasting debates and concerns regarding the potential impact of asthma on growth [3]. Possible mechanisms linking together asthma and impaired growth might include disturbance of bone maturation resulting from the atopy status [4], nutritional deprivation induced by food allergy prevention and physical inactiveness to avoid the possibility of exercise-induced bronchoconstriction [5], and potential growth retardation due to the use of glucocorticoids [6, 7].

Several studies using hospital- or community-based data have indeed suggested a negative impact of childhood asthma on growth [8–10]. However, other longitudinal studies, including a twin study, found either the null effect of asthma on height [11, 12] or a transient effect due to delayed onset of puberty [13]. The conflicting results are likely partially attributable to the methodological limitations of the previous studies (e.g., cross-sectional design [12, 14], small sample size [10, 15], and incomplete control of other potential confounders [8, 16]). Moreover, most of the existing studies focused on the height at the age of asthma diagnosis or during adolescence [10, 14]. The impact of asthma on attained adult height remains therefore largely unclear. In addition, height is highly heritable. Previous genome-wide association studies (GWAS) have shown that common variations might contribute up to 60% of the heritability for adult height [17]. It is, therefore, important to account for the genetic variance of height when comparing height between individuals with asthma and those without asthma.

To this end, leveraging detailed information on asthma diagnosis, objectively measured adult height, and the available individual-level genotype data in the UK Biobank, we conducted a cohort study to elucidate the association between childhood asthma and adult height. We specifically assessed the difference between genetically determined height and measured adult height for each participant, by comparing the rank of generated polygenetic risk scores (PRSs) for height and the rank of measured body height, and, for the first time, clarified the association between childhood asthma and adult height, beyond genetic determinants.

## Methods

### Study design

This study was based on the large prospective UK Biobank cohort described in detail elsewhere [18]. In brief, more than 500,000 individuals aged between 40 and 69 years were recruited across 22 centers in the UK between 2006 and 2010 [18]. Data on sociodemographic characteristics, lifestyle, and other health-related factors were collected using touchscreen questionnaires at recruitment, whereas anthropometric measurements and data collection on medication use (through verbal interview) were performed by nurses or trained staff during the assessment center visits [18]. In addition, blood samples were collected at baseline, and genotyping was carried out using Applied Biosystems UK BiLEVE Axiom Array and UK Biobank Axiom Array. Genotype imputation was completed using the Haplotype Reference Consortium (HRC) and UK10K haplotype resource as a reference panel using the markers that passed the UK Biobank quality control pipeline [18]. The final imputed genotype dataset contained more than 93 million autosomal single nucleotide polymorphisms (SNPs) for 488,377 individuals [18]. Follow-up data on multiple health-related outcomes for all participants were obtained through linked data from a variety of national databases, including death registries, primary, and inpatient hospital records, mapped across England, Scotland, and Wales [18]. The inpatient hospital data were deemed to cover all UK Biobank participants in January 1997 and onwards, while the primary care data were obtained from multiple general practice data system suppliers, covering approximately 45% of the UK Biobank participants [19].

**Fig. 1:**
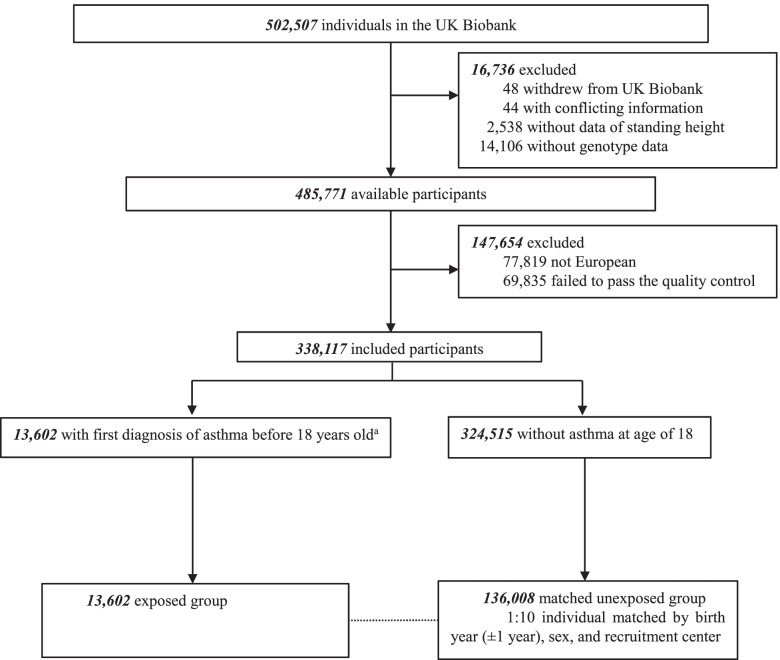
Study flowchart. ^a^Assessment of asthma is based on the ICD-10 codes (J45) recorded in the first occurrences data field

In the present study, among 502,507 individuals from the UK Biobank, we excluded participants who withdrew their consent (*n* = 48), had conflicting information (*n* = 44), or without available data on standing height (*n* = 2538) or genotype data (*n* = 14,106). In the further process of quality control for individual-level genetic data, both participants (i.e., not European, genotyping rate < 98%, outlier based on abnormal heterozygosity level, or kinship coefficient > 0.044) and variants (i.e., with a call rate < 98%, a minor allele frequency < 0.01, or deviation from Hardy-Weinberg equilibrium (*p* < 10^−6^)) that failed to pass the quality pipeline were removed. The final analytic dataset had 338,117 participants with 7,130,967 SNPs. We first compiled an exposed cohort of 13,602 individuals with a diagnosis of asthma before 18 years of age. Then, in order to have a better control of potentially important confounders including the birth cohort effect (e.g., the variation of attained height according to the year of birth) [20], we randomly selected up to 10 unexposed persons per exposed individual from the study population, who were free of asthma at the age of 18 and individually matched to the exposed individual by birth year, sex, and 22 recruitment centers (*n* = 136,008) (Fig. 1).

#### Ascertainment of asthma

With a limited coverage rate of the register data for early-life diseases, the ascertainment of asthma was mainly based on self-reported asthma in baseline questionnaires (i.e., 97.6% of all identified asthma cases). However, we additionally identified 330 incident asthma cases by the clinical diagnosis of asthma from primary care data (according to the International Classification of Diseases 10th Edition ICD-10 codes J45). To clarify the influence of age at asthma onset on adult height, we calculated age at asthma diagnosis as the time interval (in years) between the birthdate and date of first asthma diagnosis, and grouped the exposed patients into six age groups (i.e., ≤ 2, 3–4, 5–6, 7–9, 10–2, and 13–18 years). The reliability of self-reported data at baseline was assessed among 8.86% (33,224/375,037) of the study population with repeated self-reported data from follow-up surveys (i.e., first repeat assessment visit [2012–2013], imaging visit [2014], and repeat imaging visit [2019]), which showed a high agreement between these measurements, regarding both asthma diagnosis (Additional file 1: Table S1, range of consistency rates = 88.36–90.62%) and age of asthma diagnosis (as a continuous variable, range of Pearson correlation coefficients = 0.85–0.96).

#### Height outcomes

We studied three height outcomes, including attained adult height, height deviation, and height deficit. Standing height (in centimeters) measured at the first assessment center visit was used as the attained adult height. Utilizing summary statistics of a large meta-analyzed GWAS comprising 253,288 European individuals from 79 studies released by the GIANT consortium [17, 21], we generated PRS for height, as a proxy of genetically determined height. Specifically, using 1,845,024 SNPs existed in both GWAS base dataset and UK Biobank target dataset, we computed the PRS under ten *p* value thresholds (i.e., 5 × 10^−8^, 1 × 10^−6^, 1 × 10^−4^, 1 × 10^−3^, 0.05, 0.1, 0.2, 0.3, 0.4, and 0.5) in PLINK (version 1.9), and performed a regression between generated PRSs and the attained adult height [22]. The PRS with the highest Nagelkerke’s *R*^2^ (i.e., *R*^2^ = 8.8%, *p* value threshold of 1 × 10^−3^ with 6460 involved SNPs, see Additional file 1: Table S2 for details), which explained the greatest height variance, was selected for further analysis. We then quantified height deviation at the individual level as the difference between attained adult height and genetically determined height, through subtracting the individual’s rank of genetically determined height (i.e., the rank of the individual’s PRS for height within the matrix of PRSs for all study participants in the matched cohort) from their rank of attained adult height (i.e., the order of the individual’s standing height within the matrix of standing height of all study participants). We then standardized the height deviation, with a unit of increase standing for a 1% increase of height order in the study population (named as individual height deviation). We also generated a dichotomous variable “height deficit” based on the presence of negative value in individual height deviation (yes or no).

#### Covariates

Information on sociodemographic characteristics (birth year, sex, recruitment center, annual household income, educational attainment) and birth weight was collected through questionnaires at baseline. The Townsend deprivation index (TDI) was generated based on the registered postcode of family address, representing the level of area-based deprivation [23]. As the use of inhaled glucocorticoid (ICS) has been suggested to be associated with height deficit [24], we extracted information on self-reported use of ICS which was collected at baseline through verbal interview.

### Statistical analysis

For the attained adult height and individual height deviation (as continuous variables), we applied linear mixed-effect models to assess their associations with childhood asthma. We then used conditional logistic regression models to estimate the risk of the presence of height deficit in relation to childhood asthma, presented as odds ratios (ORs) with 95% confidence intervals (CIs). To explore the effect modification by age at asthma diagnosis on the studied associations, we examined and visualized age-specific estimates using the time-varying effect models described previously [25]. As a clear age-dependent risk pattern was obtained, for later analyses, we analyzed the childhood asthma by age at asthma diagnosis, first in 6 age groups [≤ 2, 3–4, 5–6, 7–9, 10–12, 13–18 years] in the main analyses and then in 3 age groups [≤ 6, 7–12, and 13–18 years] in the secondary and sensitivity analyses for maintaining better statistical power. All models were stratified by matching identifiers (birth year, sex, and recruitment center) and adjusted for birth weight (as a continuous variable), TDI (as a continuous variable), household income (< £18,000, £18,000–30,999, £31,000–51,999, £52,000–100,000, > £100,000, or unknown), and educational level (college degree, A level, O level, Certificate of Secondary Education or equivalent, National Vocation Qualifications or equivalent, other professional qualifications, or unknown). Besides the whole cohort, we did separate analyses for men and women. The difference between subgroup estimates was examined by introducing an interaction term to the regression models, and we tested the statistical significance of the trend by age at asthma diagnosis through treating the ordinal exposure variable as continuous.

We conducted stratified analyses by birth year (1936–1950, 1951–1960, and 1961–1970), birth weight (by tertile distribution: low ≤ 3.18, moderate 3.19–3.63, and high > 3.64), TDI (by tertile distribution: low ≤ − 3.28, moderate − 3.28 to − 0.96, and high > − 0.96), and annual household income (< £18,000, £18,000–52,000, and > £52,000). In addition, to address the potential effect of ICS use on the studied association, we performed subgroup analyses by the use of ICS (yes or no). With the concern of limited statistical power, these sub-analyses were only performed for 3 age groups (≤ 6, 7–12, and 13–18 years at diagnosis of asthma).

To rule out the influence of adulthood asthma, we re-calculated these estimates after excluding individuals who had an asthma diagnosis after the age of 18 (*n* = 14,515). Further, to explore to what extent the missing data on ICS use could impact our results, we applied multiple imputation where we imputed the missing values of ICS use based on all other known variables for 100 times, using the “mice” R package (imputation method = predictive mean matching) [26]. Based on each imputed dataset, we re-ran the subgroup analyses by ICS use (yes or no) and then summarized the results through calculating the average values of the 100 estimated effect sizes and their 95% CIs. Last, to test the robustness of our findings to the choice of study design, we repeated all the above analyses using a full cohort design. Namely, among the 338,117 eligible participants, 13,602 individuals with a diagnosis of asthma before 18 years of age were included in the exposed group whereas the rest were included in the unexposed group. In this analysis, the birth cohort effect was controlled by additionally adjusting for the year of birth in the regression models. All the analyses were done with the R software (version 4.0). A 2-sided *p* < 0.05 was considered statistically significant.

## Results

Of the 149,610 participants included in the analysis, 59,944 (40.07%) were born before 1950, 50,540 (33.78%) were born during 1951–1960, and 39,126 (26.15%) were born after 1961, with more men than women (57.65% vs 42.35%) (Table 1). We observed few differences in the characteristics between exposed and unexposed groups. However, with 15.7% and 29.5% of missing values on ICS use in the exposed and unexposed groups, respectively, individuals with childhood asthma seemed more likely to use ICS, compared with the matched unexposed individuals (22.56% vs 3.87%). We also showed the distribution of baseline characteristics among the study participants by age at the index date (Additional file 1: Table S3).

**Table 1:** Basic characteristic of study cohort

Characteristic	Exposed group (***n*** = 13,602)	Matched unexposed group (***n*** = 136,008)	***p***^**a**^	Total (***n*** = 149,610)
**Birth year**			0.90	
1936–1950	5427 (39.90)	54,517 (40.08)		59,944 (40.07)
1951–1960	4599 (33.81)	45,941 (33.78)		50,540 (33.78)
1961–1970	3576 (26.29)	35,550 (26.14)		39,126 (26.15)
**Sex**			1	
Women	5760 (42.35)	57,600 (42.35)		63,360 (42.35)
Men	7842 (57.65)	78,408 (57.65)		86,250 (57.65)
**Birth weight, g**			0.04	
Mean (SD)	3360 (650.00)	3350 (650.00)		3350 (650.00)
**Townsend deprivation index**			0.17	
Mean (SD)	− 1.54 (2.98)	− 1.51 (2.96)		− 1.51 (2.96)
**Educational attainment**			< 0.001	
College degree	5320 (39.11)	45,885 (33.74)		51,205 (34.23)
A level	1669 (12.27)	16,045 (11.80)		17,714 (11.84)
O levels	2728 (20.06)	29,878 (21.97)		32,606 (21.79)
CSEs	753 (5.54)	8771 (6.45)		9524 (6.37)
NVQ	875 (6.43)	9075 (6.67)		9950 (6.65)
Others	601 (4.42)	6013 (4.42)		6614 (4.42)
Unknown	1656 (12.17)	20,341 (14.96)		21,997 (14.70)
**Annual household income, £**			< 0.001	
< 18 000	2138 (15.72)	22,142 (16.28)		24,280 (16.23)
18,000–30,999	2773 (20.39)	27,929 (20.53)		30,702 (20.52)
31,000–51,999	3319 (24.40)	33,270 (24.46)		36,589 (24.46)
52,000–100,000	3084 (22.67)	28,744 (21.13)		31,828 (21.27)
> 100,000	904 (6.65)	7689 (5.65)		8593 (5.74)
Unknown	1384 (10.17)	16,234 (11.94)		17,618 (11.78)
**Recruitment center**			1	
England	12,077 (88.79)	120,759 (88.79)		132,836 (88.79)
Scotland	855 (6.29)	8550 (6.29)		9405 (6.29)
Wales	670 (4.93)	6699 (4.93)		7369 (4.93)
**Use of ICS**			< 0.001	
No	8397 (61.73)	90,567 (66.59)		98,964 (66.15)
Yes	3069 (22.56)	5265 (3.87)		8334 (5.57)
Unknown	2136 (15.70)	40,176 (29.54)		42,312 (28.28)

Data are *n* (%) or mean (SD)

*SD* standard deviation, *ICS* inhaled glucocorticoids

^a^*p* values were calculated by *T*-tests or chi-square test

Compared to the unexposed individuals, individuals with childhood asthma generally had lower attained adult height (Table 2). The magnitude of the association decreased with the increasing age at asthma diagnosis (from *β* = − 0.98 [95% CI − 1.34 to − 0.62] for asthma diagnosed at age ≤ 2 to − 0.27 [95% CI − 0.49 to − 0.05] for asthma diagnosed at 13–18 years (*p* for trend < 0.001)). The age-dependent trend seemed more prominent among men than among women (*p* for difference = 0.015).

**Table 2:** The associations between asthma diagnosed at different ages and attained adult heights

	Full cohort (***n*** = 149,610)			Men cohort (***n*** = 86,250)			Women cohort (***n*** = 63,360)			
	No. of individuals	Attained adult height (cm), mean (SD)	***β*** (95% CI)^**a**^	No. of individuals	Attained adult height (cm), mean (SD)	***β*** (95% CI)^**a**^	No. of individuals	Attained adult height (cm), mean (SD)	***β*** (95% CI)^**a**^	***p*** for sex difference^**b**^
Unexposed group	136,008	171 (9.24)	0 (ref)	78,408	176 (6.76)	0 (ref)	57,600	163 (6.27)	0 (ref)	
Exposed group, by age at asthma diagnosis (years)										
≤ 2	1150	169 (8.98)	− 0.98 (− 1.34~− 0.62)	599	175 (6.91)	− 1.42 (− 1.93~− 0.90)	551	162 (6.10)	− 0.51 (− 1.00~− 0.01)	0.012
3–4	1783	169 (9.11)	− 0.80 (− 1.09~− 0.51)	950	175 (6.51)	− 0.97 (− 1.38~− 0.56)	833	162 (6.36)	− 0.60 (− 1.01~− 0.20)	0.211
5–6	2325	171 (9.28)	− 0.38 (− 0.64~− 0.13)	1420	176 (6.73)	− 0.38 (-0.71~− 0.04)	905	163 (6.45)	− 0.40 (-0.79~− 0.02)	0.939
7–9	2650	171 (9.31)	− 0.36 (− 0.59~− 0.12)	1649	176 (6.64)	− 0.26 (− 0.57~0.05)	1001	163 (6.49)	− 0.51 (− 0.88~− 0.14)	0.310
10–12	2621	172 (9.35)	− 0.25 (− 0.49~− 0.01)	1684	177 (6.70)	− 0.13 (− 0.43~0.18)	937	163 (6.40)	− 0.49 (− 0.88~− 0.11)	0.152
13-18	3073	170 (9.50)	− 0.27 (− 0.49~− 0.05)	1540	177 (6.90)	− 0.04 (− 0.37~0.28)	1533	163 (6.30)	− 0.52 (− 0.82~− 0.22)	0.035
*p* for trend^c^			< 0.001			0.005			< 0.001	0.015

*CI* confidence interval

^a^*β* were estimates derived from linear mixed-effect models stratified by matching identifiers (birth year, sex, and recruitment center) and adjusted for birth weight, Townsend deprivation index, education level, and annual household income

^b^The statistical significance of the difference between sexes was assessed by including an interaction term in the linear model

^c^*p* values for dose-response trends were calculated by fitting ordinal exposure variables as continuous terms into the linear models

The time-varying analyses indicated that there was a significant association along with a clear trend of decreasing magnitude of the association between asthma and adult height with increasing age at asthma diagnosis, primarily for asthma diagnosed before age 8 (Additional file 1: Fig. S1). Likewise, in age-specific subgroup analyses, we found an increasing trend of height deviation with younger age at asthma diagnosis (*p* for trend = 0.022), with the strongest association observed for asthma diagnosed before 4 years of age (− 2.57 [95% CI − 4.14 to − 1.00] and − 2.80 [95% CI − 4.06 to − 1.54] for asthma diagnosed at age ≤ 2 and 3–4 years, respectively) (Table 3). The height deviation became less prominent for asthma diagnosed afterwards and lost statistical significance for asthma diagnosed after age 6. Similar results were obtained when studying the presence of height deficit. Half of the unexposed individuals were classified as having a height deficit. This proportion increased to more than 55% among individuals with asthma diagnosed at ≤ 4. This corresponded to an OR of 1.21 (95% CI 1.04 to 1.40) and 1.15 (95% CI 1.02 to 1.29) for height deficit in relation to an asthma diagnosis at ≤ 2 and 3–4 years, respectively (Table 3).

**Table 3:** The associations of asthma with height deviation and deficit

	**Full cohort (*n*** **= 149,610)**			**Men cohort (*n*** **= 86,250)**			**Women cohort (*n*** **= 63,360)**			
***Outcome: individual height deviation (%)***^***c***^										
	**No. of individuals**	**Mean (SD)**	***β*** **(95% CI)**^**a**^	**No. of individuals**	**Mean (SD)**	***β*** **(95% CI)**^**a**^	**No. of individuals**	**Mean (SD)**	***β*** **(95% CI)**^**a**^	***p*** **for sex difference**^**e**^
Unexposed group	136,008	0.06 (34.6)	0 (ref)	78,408	0.07 (31.3)	0 (ref)	57,600	0.11 (22.7)	0 (ref)	
Exposed group, by age at asthma diagnosis (years)										
≤ 2	1150	− 5.56 (34.8)	− 2.57 (− 4.14~− 1.00)	599	− 5.29 (32.0)	− 4.50 (− 6.93~− 2.06)	551	− 2.60 (22.9)	− 1.82 (− 3.66~0.02)	0.119
3–4	1783	− 5.11 (34.4)	− 2.80 (− 4.06~− 1.54)	950	− 4.65 (30.0)	− 4.23 (− 6.16~− 2.29)	833	− 3.36 (22.8)	− 2.76 (− 4.26~− 1.26)	0.119
5–6	2325	0.25 (34.4)	− 1.14 (− 2.24~− 0.03)	1420	− 1.76 (30.9)	− 1.95 (− 3.54~− 0.36)	905	− 1.47 (23.5)	− 1.22 (− 2.66~0.22)	0.505
7–9	2650	1.85 (34.2)	− 0.78 (− 1.81~0.26)	1649	− 0.33 (31.5)	− 1.59 (− 3.06~− 0.11)	1001	− 0.34 (23.2)	− 1.03 (− 2.40~0.35)	0.585
10–12	2621	3.35 (34.5)	− 0.31 (− 1.36~0.73)	1684	0.91 (31.3)	− 0.76 (− 2.33~0.70)	937	− 0.13 (23.5)	− 0.41 (− 1.82~1.01)	0.745
13–18	3073	− 2.32 (35.5)	− 0.06 (− 1.02~0.90)	1540	2.18 (31.6)	0.80 (− 0.72~2.33)	1533	− 0.18 (23.4)	− 1.28 (− 2.40~− 0.17)	0.031
*p* for trend^f^			0.022			0.027			< 0.001	0.404
***Outcome: height deficit (yes or no)***^***d***^										
	**No. of individuals with height deficit/no. of total individuals (%)**		**OR (95% CI)**^**b**^	**No. of individuals with height deficit/no. of total individuals (%)**		**OR (95% CI)**^**b**^	**No. of individuals with height deficit/no. of total individuals (%)**		**OR (95% CI)**^**b**^	***p*** **for sex difference**^**e**^
Unexposed group	67,291/136,008 (49.48)		1(ref)	38,824/78,408 (49.52)		1(ref)	28,493/57,600 (49.47)		1(ref)	
Exposed group, by age at asthma diagnosis (years)										
≤ 2	642/1150 (55.83)		1.21 (1.04~1.40)	347/599 (57.93)		1.38 (1.16~1.64)	298/551 (54.08)		1.15 (0.96~1.38)	0.154
3–4	983/1783 (55.13)		1.15 (1.02~1.29)	536/950 (56.42)		1.30 (1.13~1.49)	464/833 (55.70)		1.22 (1.05~1.41)	0.538
5–6	1125/2325 (48.39)		1.05 (0.94~1.16)	759/1420 (53.45)		1.18 (1.05~1.32)	472/905 (52.15)		1.07 (0.93~1.23)	0.288
7–9	1236/2650 (46.64)		1.02 (0.93~1.12)	836/1649 (50.70)		1.12 (1.01~1.24)	506/1001 (50.55)		1.07 (0.94~1.23)	0.597
10–12	1194/2621 (45.56)		1.03 (0.94~1.13)	834/1684 (49.52)		1.09 (0.99~1.21)	453/937 (48.35)		1.01 (0.88~1.16)	0.382
13–18	1606/3073 (52.26)		0.98 (0.90~1.07)	731/1540 (47.47)		0.98 (0.88~1.09)	749/1533 (48.86)		1.07 (0.96~1.19)	0.256
*p* for trend^f^			0.287			< 0.001			0.054	0.065

*SD* standard deviation, *CI* confidence interval, *OR* odds ratio

^a^*β* were estimates derived from the linear mixed-effect models stratified by matching identifiers (birth year, sex, and recruitment center) and adjusted for birth weight, Townsend deprivation index, education level, and annual household income

^b^ORs (95% CI) were derived from conditional logistic regression models stratified by matching identifiers (birth year, sex, and recruitment center) and adjusted for birth weight, Townsend deprivation index, education level, and annual household income

^c^Individual height deviation (%): calculated by (rank of attained adult height-rank of genetically determined height)/149,610 × 100

^d^Height deficit: for an individual, rank of attained adult height < rank of genetically determined height (yes or no)

^e^The statistical significance of the difference between sexes was assessed by including an interaction term in the linear and logistic regression models

^f^*p* values for dose-response trends were calculated by fitting ordinal exposure variables as continuous terms into the linear and logistic models

Analysis of asthma diagnosis at ≤ 6, 7–12, or 13–18 years yielded similar results, with significant individual height deviation and height deficit observed among individuals with asthma diagnosed at age 6 or earlier (Additional file 1: Table S4). The stratified analyses revealed that the associations were not substantially modified by birth weight or TDI; weaker associations were noted among individuals born after 1961, compared to those born before then, and those with higher annual household income, compared with those with lower annual household income (Additional file 1: Table S5). The result pattern was similar when separately analyzing asthma with or without ICS use, although the estimates were consistently stronger among asthma individuals who used ICS (Table 4).

**Table 4:** Associations between asthma and height by use of inhaled glucocorticoids (ICS)

	**ICS use (*n*** **= 24,864)**			**No ICS use (*n*** **= 68,493)**			
***Outcome: attained adult height (cm)***							
	**No. of individuals**	**Mean (SD)**	**β (95% CI)**^**a**^	**No. of individuals**	**Mean (SD)**	***β*** **(95% CI)**^**a**^	***p*** **for difference**^**e**^
Unexposed group	21,795	169 (9.26)	0 (ref)	60,096	170 (9.19)	0 (ref)	
Exposed group, by age at asthma diagnosis (years)							
≤ 6	1325	169 (9.47)	− 0.85 (− 1.19~− 0.51)	3199	170 (9.13)	− 0.59 (− 0.81~− 0.37)	0.208
7–12	1046	171 (9.55)	− 0.61 (− 0.99~− 0.23)	3269	171 (9.31)	− 0.28 (− 0.5~− 0.07)	0.141
13–18	698	168 (9.63)	− 0.41 (− 0.87~0.06)	1929	170 (9.33)	− 0.17 (− 0.45~0.11)	0.382
*p* for trend^f^			< 0.001			< 0.001	0.035
***Outcome: individual height deviation (%)***^***c***^							
	**No. of individuals**	**Mean (SD)**	***β*** **(95% CI)**^**a**^	**No. of individuals**	**Mean (SD)**	***β*** **(95% CI)**^**a**^	***p*** **for difference**^**e**^
Unexposed group	21,795	− 3.75 (34.6)	0 (ref)	60,096	− 2.93 (34.6)	0 (ref)	
Exposed group, by age at asthma diagnosis (years)							
≤ 6	1325	− 4.16 (34.3)	− 2.32 (− 3.82~− 0.82)	3199	− 3.63 (34.5)	− 1.68 (− 2.64~− 0.72)	0.481
7–12	1046	0.38 (33.3)	− 1.44 (− 3.12~0.24)	3269	1.31 (35.1)	− 0.13 (− 1.09~0.82)	0.185
13–18	698	− 6.57 (37.3)	− 0.88 (− 2.92~1.16)	1929	− 2.55 (35.1)	0.76 (− 0.47~1.98)	0.119
*p* for trend^f^			0.014			0.991	0.243
***Outcome: height deficit (yes or no)***^***d***^							
	**No. of individuals with height deficit/no. of total individuals (%)**		**OR (95% CI)**^**b**^	**No. of individuals with height deficit/no. of total individuals (%)**		**OR (95% CI)**^**b**^	***p*** **for difference**^**e**^
Unexposed group	11,839 (54.32)		1(ref)	31,863 (53.02)		1(ref)	
Exposed group, by age at asthma diagnosis (years)							
≤ 6	732 (55.25)		1.29 (1.12–1.48)	1706 (53.33)		1.10 (1.01–1.21)	0.060
7–12	508 (48.57)		1.04 (0.89–1.21)	1563 (47.81)		1.02 (0.93–1.11)	0.830
13–18	394 (56.45)		0.98 (0.81–1.19)	1025 (53.14)		0.98 (0.87–1.10)	1.00
*p* for trend^f^			0.215			0.815	0.059

*SD* standard deviation, *CI* confidence interval, *OR* odds ratio, *ICS* inhaled glucocorticoids

^a^*β* were estimates derived from the linear mixed-effect models stratified by matching identifiers (birth year, sex, and recruitment center) and adjusted for birth weight, Townsend deprivation index, education level, and annual household income

^b^ORs (95% CI) were derived from the conditional logistic regression models stratified by matching identifiers (birth year, sex, and recruitment center), and adjusted for birth weight, Townsend deprivation index, education level, and annual household income

^c^Individual height deviation (%): calculated by (rank of attained adult height-rank of genetically determined height)/149,610 × 100

^d^Height deficit: for an individual, rank of attained adult height < rank of genetically determined height (yes or no)

^e^The statistical significance of the difference between sexes was assessed by including an interaction term in the linear and logistic regression models

^f^*p* values for dose-response trends were calculated by fitting ordinal exposure variables as continuous terms into the linear and logistic models

The sensitivity analysis indicated that stronger associations were found when unexposed individuals with asthma diagnosed after the age of 18 were removed (Additional file 1: Table S6). Also, the ICS use-specific HRs remained unchanged after multiple imputation of missing values of ICS use (Additional file 1: Table S7). In addition, the alternative use of a full cohort design led to largely similar estimates as those obtained in the main analyses (Additional file 1: Table S8).

## Discussion

In this community-based matched cohort study based on the UK Biobank, we found that individuals with childhood asthma were at risk for having lower attained adult height compared to individuals without such an experience, after controlling for multiple important factors, such as birth year and weight, and socioeconomic status. Importantly, we considered the individual height deviation, by quantifying the changes in height order between genetically determined height and attained adult height and performed separate analyses for asthma patients diagnosed at different ages. Our findings, for the first time, demonstrated a notable negative association between childhood asthma, primarily before age 7, and adult height, after consideration of genetic components of height. Furthermore, as height deficit was also observed among asthma individuals not using ICS, the results suggest that multiple factors might have attributed to the height loss observed among asthma patients.

Although previous studies have reported a growth retarding effect of childhood asthma [27], the evidence on the attained adult height is inclusive. Consistent with our findings, several large-scale studies based on military conscription records or medical birth registers suggested a negative impact of childhood asthma on adult height, even after taking into consideration of ICS use [16, 28]. Other studies failed however to find an association [11, 12, 29]. The methodological heterogeneities between studies, including the inclusion of children with different disease severity of asthma and varying sample sizes, might have contributed to the discrepant findings of previous work. Self-reported asthma diagnosis, although widely used in large population- or community-based surveys [9, 13], might lead to concern of recall bias whereas the clinical diagnosis of asthma identified through healthcare register data might suffer from the often incomplete coverage of the registers and the possibility of delayed registration [8, 10]. Furthermore, genetic and other familial factors are important determinants of adult height. Because these are difficult to assess, they are often overlooked when investigating the impact of a specific condition on height. Prior efforts in overcoming genetic confounding were very limited, with only one study featured by the use of twin-based data [13]. Based on self-reported data on asthma diagnosis and height from 2658 Swedish twins, no overall height difference was observed in twin pairs discordant for asthma, although the study, similar to the present study, showed indeed that asthma had a greater impact on the height of twin boys than on twin girls [13]. The limited sample size (e.g., only 826 same-sex twin pairs involved) might have rendered difficulties in assessing the reliability of these results.

More recently, height PRS, derived by multiple genetic variants which are known to explain a large proportion of the heritability of height, has been used as a proxy for target height in epidemiologic studies, providing a way to address the genetic confounding between height and other traits [30]. Consequently, using the novel approach where we estimated the individual’s deviation from their target height by comparing the person’s rank of attained adult height to the rank of genetically determined height, we for the first time demonstrated the clear impact of childhood asthma on adult height, irrespective of one’s genetic contribution on height. These findings underscore therefore the need for surveillance on the growth of children with asthma.

Our finding that asthma diagnosed at an earlier age was associated with greater adult height deficit is in line with previous reports, although investigations on the impact of age at asthma onset were scarce. For instance, a study of Swedish females with broader age groups (0–8, 9–15, and 16+ years) demonstrated that among girls, the younger age of hospitalization for asthma was associated with a shorter adult height, with the largest height loss observed among girls hospitalized for asthma at the youngest age [28]. Our analyses by sex obtained similar results, suggesting a stronger association between childhood asthma and height among men than among women [13]. Together, our sex- and age-specific analyses expend this knowledgebase by highlighting a 2–3% reduction of height order among men and women with asthma diagnosed before the age of 7, whereas asthma diagnosed at later ages has little impact on height among women.

The underlying mechanisms linking asthma to reduced adult height remain unclear. Previous studies speculated that early-life asthma might result in a delayed puberty, through dysregulation of sex hormone production, which might explain height deficit [31, 32]. In addition, experimental studies have reported various mediators related to the eosinophilia process of asthma, such as prostaglandins and the platelet-activating factor, known to influence local growth factor prostaglandin E(2) synthesis in the osteoblasts, which may consequently have a negative impact on collagen synthesis and formation of new bone [4]. In the present study, we found the most pronounced risk increase of height deviation/deficit for individuals with asthma diagnosed during the pre-puberty stage, supporting the notion that the impact of childhood asthma or ICS use on puberty plays an important role in the development disturbances. Impaired nocturnal growth hormone secretion and other endocrine malfunction in relation to puberty were indeed observed among children with asthma [33]. Other proposed mechanisms could be that several asthma-related symptoms and disorders in severe asthma might lead to growth restriction, such as chronic hypoxia, diminished lung function, chronic infection, and sleep disturbance [34]. In addition, the growth retarding effect of ICS has been confirmed by a large number of studies [35]. Nevertheless, as a similar risk pattern was observed among the asthma individuals not using ICS in the present study, our results suggest that asthma alone could also have a negative impact on height. Lastly, behavior-related factors, such as physical inactivity [36] and nutrition deficiency [33], often observed among asthma patients, might have also contributed to the height loss.

The major merit of our study is the combined use of objectively measured adult height and PRS for height (as a proxy for genetically determined height), which enabled the control for genetic confounding due to heritability (i.e., genetic heterogeneity) of height. Also, the application of a matched cohort design addressed the concern of the birth cohort effect, which is considered critical in our analyses as the average adult height as well as the societal awareness and diagnosis criteria of asthma have changed over the study period [20, 37]. Furthermore, thanks to the large sample size of the UK Biobank, we were able to categorize the diagnosis age of asthma into six groups. This facilitated the discussion of the differential associations of age at asthma onset with height impairment, shedding light on the potential underlying mechanisms including impaired growth velocity. Indeed, we found the degree of height loss to decrease with the increase of age at asthma diagnosis. The availability of enriched phenotypic and self-reported data allowed the consideration of a wide range of covariables, especially sociodemographic status and birth weight, both of which might have played a role in the association between asthma and height [38], as well as the self-reported use of ICS.

Our study also has several limitations. First, the fact that 97.6% of the asthma cases were ascertained through self-reported data might have led to misclassification of exposure to some extent, i.e., severer and persist asthma cases might be more likely to be reported than milder cases. Also, given the likely lower certainty of self-reported data on the diagnosis date of asthma, the results of age-specific analyses need to be interpreted with caution, although this is unlikely to explain entirely the observed clear trend of decreasing impact of asthma on adult height by increasing age at asthma diagnosis. Furthermore, in a small proportion of the study population with repeated measurements, we indeed find a good agreement between the self-reported asthma data at baseline and in follow-up surveys. Second, besides the use of ICS, we had no information on the symptom or severity of asthma. Therefore, whether the observed adult height deficit in asthma individuals could be modified by the severity of the disease needs to be addressed in future studies. Third, we cannot rule out the possibility of residual confounding from unmeasured factors. For instance, it is important to note that the lack of information about early life conditions precludes the possibility to explore their roles in the observed associations. Also, the effect of medications other than ICS was not discussed in this study. Future investigations with detailed data on childhood-to-adulthood lifestyle factors and medication use are warranted. Finally, we had relatively limited statistical power in the analysis of diagnosis age for asthma.

## Conclusions

Our analyses of a large matched cohort study based on the UK Biobank provide evidence that childhood asthma was associated with a lower adult height. These associations remain after the careful consideration of genetic variance of height but are stronger among men and likely mainly attributable to the impact of early-onset asthma. The observation of height deficit among both asthma individuals with and without ICS use suggests other mechanisms might play an important role in the asthma-related height deficit, which needs to be explored in future studies.

## Supplementary Information

**Additional file 1: Figure S1**. Age-varying associations between childhood asthma and individual height deviation (%) (Panel A), and the presence of height deficit (yes or no) (Panel B). **Table S1**. Agreement of self-reported data on asthma diagnosis between baseline and repeat measurements. **Table S2**. Associations between height PRSs and the height at different *p* value thresholds. **Table S3**. Baseline characteristic of study participants, by age at the index date. **Table S4**. The associations between asthma and attained adult height / height change (height deviation and deficit) at individual. **Table S5**. Associations between asthma and attained adult height /individual height change (height deviation and deficit) stratified by different characteristics. **Table S6.** Sensitivity analysis for the association between asthma and attained adult height / height change (height deviation and deficit) by removing unexposed individuals with asthma diagnosed after age of 18. **Table S7**. Associations between asthma and height by use of inhaled glucocorticoids (ICS), after multiple imputation for the missing values of ICS use. **Table S8**. The associations between asthma and attained adult height / height change (height deviation and deficit), based on analysis of a full cohort design.

## Data Availability

Data from the UK Biobank (http://www.ukbiobank.ac.uk/) are available to all researchers upon making an application. This research was done using the UK Biobank Resource under Application 54803 (approved on October 29, 2019).

## Abbreviations

CI: Confidence interval
GWAS: Genome-wide association studies
HRC: Haplotype Reference Consortium
ICD-10: International Classification of Diseases 10th Edition
ICS: Inhaled glucocorticoid
OR: Odds ratio
PRSs: Polygenetic risk scores
SNPs: Single nucleotide polymorphisms
TDI: Townsend deprivation index
