# Supplementary material for: The influence of childhood asthma on adult height: evidence from the UK Biobank
Source: BMC Med. 2022 Mar 22;20:94. doi: 10.1186/s12916-022-02289-1 (PMC8939112; doi:10.1186/s12916-022-02289-1)
Supplement: Supplementary file 1 — Additional file 1: Figure S1. Age-varying associations between childhood asthma and individual height deviation (%) (Panel A), and the presence of height deficit (yes or no) (Panel B). Table S1. Agreement of self-reported data on asthma diagnosis between baseline and repeat measurements. Table S2. Associations between height PRSs and the height at different p value thresholds. Table S3. Baseline characteristic of study participants, by age at the index date. Table S4. The associations between asthma and attained adult height / height change (height deviation and deficit) at individual. Table S5. Associations between asthma and attained adult height /individual height change (height deviation and deficit) stratified by different characteristics. Table S6. Sensitivity analysis for the association between asthma and attained adult height / height change (height deviation and deficit) by removing unexposed individuals with asthma diagnosed after age of 18. Table S7. Associations between asthma and height by use of inhaled glucocorticoids (ICS), after multiple imputation for the missing values of ICS use. Table S8. The associations between asthma and attained adult height / height change (height deviation and deficit), based on analysis of a full cohort design. [file 12916_2022_2289_MOESM1_ESM.docx]

**The influence of childhood asthma on adult height: evidence from the UK Biobank**

**Additional File 1**

Wenwen Chen; Huanzhen Yang; Can Hou; Yajing Sun; Yanan Shang; Yu Zeng; Yao Hu; Yuanyuan Qu; Jianwei Zhu; Fang Fang; Donghao Lu; Huan Song

**Content**

**Figure S1** Age-varying associations between childhood asthma and individual height deviation (%) (Panel A), and the presence of height deficit (yes or no) (Panel B)

**Table S1** Agreement of self-reported data on asthma diagnosis between baseline and repeat measurements

**Table S2** Associations between height PRSs and the height at different p value thresholds

**Table S3** Baseline characteristic of study participants, by age at the index date

**Table S4** The associations between asthma and attained adult height / height change (height deviation and deficit) at individual

**Table S5** Associations between asthma and attained adult height /individual height change (height deviation and deficit) stratified by diﬀerent characteristics

**Table S6** Sensitivity analysis for the association between asthma and attained adult height / height change (height deviation and deficit) by removing unexposed individuals with asthma diagnosed after age of 18

**Table S7** Associations between asthma and height by use of inhaled glucocorticoids (ICS), after multiple imputation for the missing values of ICS use

**Table S8** The associations between asthma and attained adult height / height change (height deviation and deficit), based on analysis of a full cohort design


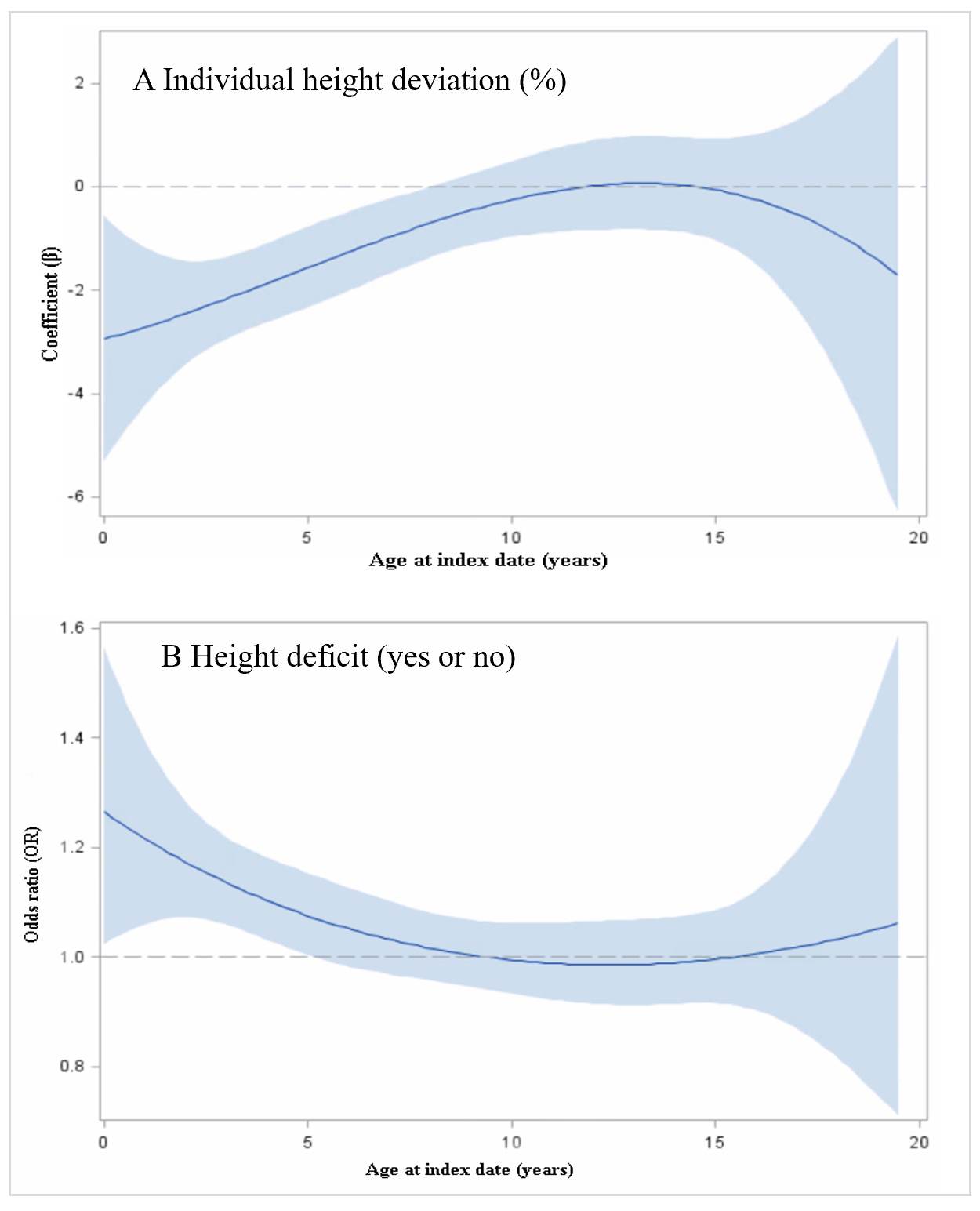


**Figure S1** Age-varying associations between childhood asthma and individual height deviation (%) (Panel A), and the presence of height deficit (yes or no) (Panel B)

*The age-varying coefficients (βs) were derived from time-varying effect models. These models applied P-spline approach to split complex functions into numerous segments, each of which was estimated with a polynomial model. All models were adjusted for birth year, sex, recruitment center, birth weight, Townsend deprivation index, education level, and annual household income.

**Table S1** Agreement of self-reported data on asthma diagnosis between baseline and repeat measurements

| Instance 0:  Baseline assessment visit (2006-2010) | Instance 1: First repeat assessment visit (2012-2013) | | | Instance 2: Imaging visit (2014) | | | Instance 3: Repeat imaging visit (2019) | | |
| --- | --- | --- | --- | --- | --- | --- | --- | --- | --- |
| ***The presence of asthma diagnosis (≤ 18 years)*** | | | | | | | | | |
|  | **Yes** | **No** | **Unknown** | **Yes** | **No** | **Unknown** | **Yes** | **No** | **Unknown** |
| **Yes** | *561* | 78 | 42 | *1044* | 153 | 80 | *12* | 2 | 5 |
| **No** | 82 | *12031* | 520 | 290 | *19843* | 740 | 5 | *375* | 11 |
| **Unknown** | 14 | 446 | 121 | 43 | 1229 | 131 | 1 | 25 | 2 |
| Consistency rate ^a^ | 12592/13895 (90.62%) | | | 20887/23553 (88.68%) | | | 387/438 (88.36%) | | |
| ***Age at asthma diagnosis (as continuous variable)*** | | | | | | | | | |
| Pearson correlation coefficient | 0.899 | | | 0.851 | | | 0.957 | | |
| ***Age of asthma diagnosis in groups (years)*** | | | | | | | | | |
|  | **≤6** | **7-12** | **13-18** | **≤6** | **7-12** | **13-18** | **≤6** | **7-12** | **13-18** |
| **≤6** | *214* | 28 | 1 | *378* | 68 | 2 | *5* | 0 | 0 |
| **7-12** | 39 | *175* | 16 | 79 | *303* | 33 | 1 | *4* | 1 |
| **13-18** | 2 | 13 | *73* | 6 | 34 | *141* | 0 | 0 | *1* |
| Consistency rate ^b^ | 462/561 (82.35%) | | | 822/1044 (78.74%) | | | 10/12 (83.33%) | | |

^a^. No. of individual with same asthma diagnose (*≤ 18 years*) (both yes or both no)/No. of individual participated in both assessments.

^b^. No. of individual with same age group of asthma diagnose/No. of individual with asthma diagnosis (*≤ 18 years*) in both assessments.

**Table S2** Associations between height PRSs and the height at different p value thresholds

| Pt | BETA^*^ | SE | R^2^ | P |
| --- | --- | --- | --- | --- |
| Pt5e-08 | 2.3969 | 0.0101 | 6.67% | 0 |
| Pt1e-06 | 2.4930 | 0.0100 | 7.21% | 0 |
| Pt1e-04 | 2.6949 | 0.0099 | 8.37% | 0 |
| Pt0.001 | 2.7823 | 0.0099 | 8.82% | 0 |
| Pt0.05 | 2.7657 | 0.0102 | 8.26% | 0 |
| Pt0.1 | 2.7378 | 0.0103 | 7.99% | 0 |
| Pt0.2 | 2.7091 | 0.0104 | 7.73% | 0 |
| Pt0.3 | 2.6928 | 0.0105 | 7.58% | 0 |
| Pt0.4 | 2.6827 | 0.0105 | 7.51% | 0 |
| Pt0.5 | 2.6782 | 0.0105 | 7.47% | 0 |

Pt, p value threshold; SE, stand error; R^2^, Nagelkerke’s squared (R square).

**^*^**Beta (per standard deviation increase in the corresponding PRS) were derived from linear regression models adjusting for age, sex, genotyping array, and ancestry principal components.

**Table S3** Baseline characteristic of study participants, by age at the index date^a^

| **Characteristics** | ≤ 6 years | | 7-12 years | | 13-18 years | |
| --- | --- | --- | --- | --- | --- | --- |
|  | Exposed group | Matched unexposed group | Exposed group | Matched unexposed group | Exposed group | Matched unexposed group |
|  | (n=5258) | (n=52575) | (n=5271) | (n=52703) | (n=3073) | (n=30730) |
| **Birth year** |  |  |  |  |  |  |
| 1936-1950 | 2423 (46.08) | 24215 (46.06) | 2000 (37.94) | 20192 (38.31) | 1004 (32.67) | 10110 (32.90) |
| 1951-1960 | 1759 (33.45) | 17713 (33.69) | 1851 (35.12) | 18325 (34.77) | 989 (32.18) | 9903 (32.23) |
| 1961-1970 | 1076 (20.46) | 10647 (20.25) | 1420 (26.94) | 14186 (26.92) | 1080 (35.14) | 10717 (34.87) |
| **Sex** |  |  |  |  |  |  |
| Female | 2289 (43.53) | 22890 (43.54) | 1938 (36.77) | 19380 (36.77) | 1533 (49.89) | 15330 (49.89) |
| Male | 2969 (56.47) | 29685 (56.46) | 3333 (63.23) | 33323 (63.23) | 1540 (50.11) | 15400 (50.11) |
| **Birth weight, grams** |  |  |  |  |  |  |
| Mean (SD) | 3350 (658) | 3340 (661) | 3400 (638) | 3360 (655) | 3310 (644) | 3340 (642) |
| **Townsend deprivation index** |  |  |  |  |  |  |
| Mean (SD) | -1.58 (2.93) | -1.55 (2.94) | 1.58 (2.95) | 1.49 (2.97) | -1.42 (3.10) | -1.46 (2.97) |
| **Educational attainment** |  |  |  |  |  |  |
| College degree | 1964 (37.35) | 17364 (33.03) | 2209 (41.91) | 18015 (34.18) | 1147 (37.33) | 10506 (34.19) |
| A level | 623 (11.85) | 6041 (11.49) | 633 (12.01) | 6288 (11.93) | 413 (13.44) | 3716 (12.09) |
| O levels | 1046 (19.89) | 11365 (21.62) | 1037 (19.67) | 11440 (21.71) | 645 (20.99) | 7073 (23.02) |
| CSEs | 275 (5.23) | 3083 (5.86) | 285 (5.41) | 3497 (6.64) | 193 (6.28) | 2191 (7.13) |
| NVQ | 361 (6.87) | 3584 (6.82) | 324 (6.15) | 3653 (6.93) | 190 (6.18) | 1838 (5.98) |
| Other | 258 (4.91) | 2473 (4.70) | 201 (3.81) | 2251 (4.27) | 142 (4.62) | 1289 (4.19) |
| Unknown | 731 (13.90) | 8665 (16.48) | 582 (11.04) | 7559 (14.34) | 343 (11.16) | 4117 (13.40) |
| **Annual household income, £** |  |  |  |  |  |  |
| <18 000 | 906 (17.23) | 9070 (17.25) | 752 (14.27) | 8232 (15.62) | 480 (15.62) | 4840 (15.75) |
| 18 000-30 999 | 1090 (20.73) | 11113 (21.14) | 1053 (19.98) | 10684 (20.27) | 630 (20.50) | 6132 (19.95) |
| 31 000-51 999 | 1300 (24.72) | 12553 (23.88) | 1268 (24.06) | 13042 (24.75) | 751 (24.44) | 7675 (24.98) |
| 52 000-100 000 | 1059 (20.14) | 10422 (19.82) | 1337 (25.37) | 11580 (21.97) | 688 (22.39) | 6742 (21.94) |
| >100 000 | 306 (5.82) | 2760 (5.25) | 391 (7.42) | 3175 (6.02) | 207 (6.74) | 1754 (5.71) |
| Unknown | 597 (11.35) | 6657 (12.66) | 470 (8.92) | 5990 (11.37) | 317 (10.32) | 3587 (11.67) |
| **Recruitment center** |  |  |  |  |  |  |
| England | 4625 (87.96) | 46245 (87.96) | 4711 (89.38) | 47104 (89.38) | 2741 (89.20) | 27410 (89.20) |
| Scotland | 341 (6.49) | 3410 (6.49) | 313 (5.94) | 3130 (5.94) | 201 (6.54) | 2010 (6.54) |
| Wales | 292 (5.55) | 2920 (5.55) | 247 (4.69) | 2469 (4.68) | 131 (4.26) | 1310 (4.26) |
| **Use of ICS** |  |  |  |  |  |  |
| No | 3199 (60.84) | 35669 (67.84) | 3269 (62.02) | 34576 (65.61) | 1929 (62.77) | 20322 (66.13) |
| Yes | 1325 (25.20) | 2160 (4.11) | 046 (19.84) | 1980 (3.76) 1 | 698 (22.71) | 1125 (3.66) |
| Unknown | 734 (13.96) | 14746 (28.05) | 956 (18.14) | 16147 (30.64) | 446 (14.51) | 9283 (30.21) |

Data are n (%) or mean (SD), ICS = inhaled glucocorticoids. SD= standard deviation**.**

a. The index date was the date of asthma diagnosis for the exposed persons with asthma and their individually matched unexposed persons.

**Table S4** The associations between asthma and attained adult height / height change (height deviation and deficit) at individual

|  | Full cohort (n=149610) | | | Male cohort (n=86250) | | | Female cohort (n=63360) | | |
| --- | --- | --- | --- | --- | --- | --- | --- | --- | --- |
|  |  |  |  |  |  |  |  |  |  |
| ***Outcome: Attained adult height(centimeter)*** | | | | | | | | | |
|  | **No. of individuals** | **Mean (SD)** | **β (95% CI)^a^** | **No. of individuals** | **Mean (SD)** | **β (95% CI)^a^** | **No. of individuals** | **Mean (SD)** | **β (95% CI)^a^** |
| Unexposed group | 136608 | 171 (9.24) | 0 (ref) | 78408 | 176 (6.76) | 0 (ref) | 57600 | 163 (6.27) | 0 (ref) |
| Exposed group, by age at asthma diagnosis (years) | | | |  |  |  |  |  |  |
| ≤ 6 | 5258 | 170 (9.20) | -0.66 (-0.83~-0.48) | 2969 | 176 (6.72) | -0.78 (-1.01~-0.54) | 2289 | 163 (6.33) | -0.50 (-0.75~-0.26) |
| 7-12 | 5271 | 172 (9.33) | -0.30 (-0.47~-0.13) | 3333 | 177 (6.67) | -0.19 (-0.42~-0.03) | 1938 | 163 (6.44) | -0.50 (-0.77~-0.24) |
| 13-18 | 3073 | 170 (9.50) | -0.27 (-0.49~-0.05) | 1540 | 177 (6.90) | -0.04 (-0.37~0.28) | 1533 | 163 (6.30) | -0.52 (-0.82~-0.22) |
| ***Outcome: Individual height deviation (%) ^c^*** | | | | | | |  | | |
|  | **No. of individuals** | **Mean (SD)** | **β (95% CI)^a^** | **No. of individuals** | **Mean (SD)** | **β (95% CI)^a^** | **No. of individuals** | **Mean (SD)** | **β (95% CI)^a^** |
| Unexposed group | 136608 | -0.06 (34.6) | 0 (ref) | 78408 | 0.07 (31.3) | 0 (ref) | 57600 | 0.11 (22.7) | 0 (ref) |
| Exposed group, by age at asthma diagnosis (years) | | | |  |  |  |  |  |  |
| ≤ 6 | 5258 | 2.84 (34.6) | -2.02 (-2.76~-1.27) | 2969 | -3.40 (30.9) | -3.19 (-4.30~-2.08) | 2289 | -2.43 (23.1) | -1.92 (-2.84~-1.01) |
| 7-12 | 5271 | -2.60 (34.4) | -0.55 (-1.29~0.19) | 3333 | 0.30 (31.4) | -1.17 (-2.22~-0.12) | 1938 | -0.11 (23.3) | -0.73 (-1.72~0.27) |
| 13-18 | 3073 | 2.32 (35.5) | -0.06 (-1.02~0.90) | 1540 | 2.18 (31.6) | 0.80 (-0.72~2.33) | 1533 | -0.18 (23.4) | -1.28 (-2.40~-0.17) |
| ***Outcome: Height deficit*** ***(yes or no) ^d^*** | | | | | | | | | |
|  | **No. of individuals with height deficit/No. of total individuals (%)** | | **OR (95% CI)^b^** | **No. of individuals with height deficit/No. of total individuals (%)** | | **OR (95% CI)^b^** | **No. of individuals with height deficit/No. of total individuals (%)** | | **OR (95% CI)^b^** |
|  |  |  |  |  |  |  |  |  |  |
| Unexposed group | 67291/136608 (49.48) | | 1(ref) | 38824/78408 (49.52) | | 1(ref) | 28493/57600 (49.47) | | 1(ref) |
| Exposed group, by age at asthma diagnosis (years) | | | |  | |  |  | |  |
| ≤ 6 | 2750/5258 (52.30) | | 1.11 (1.04-1.19) | 1642/2969 (55.30) | | 1.25 (1.16-1.35) | 1234/2289 (53.91) | | 1.14 (1.04-1.25) |
| 7-12 | 2430/5271 (46.10) | | 1.02 (0.96-1.10) | 1670/3333 (50.11) | | 1.10 (1.03-1.19) | 959/1938 (49.48) | | 1.04 (0.94-1.15) |
| 13-18 | 1606/3073 (52.26) | | 0.98 (0.90-1.07) | 731/1540 (47.47) | | 0.98 (0.88-1.09) | 749/1533 (48.86) | | 1.07 (0.96-1.19) |

^a^ β were estimate derived from linear mixed-effect models stratified by matching identifiers (birth year, sex and recruitment center), and adjusted for birth weight, Townsend deprivation index, education level, and annual household income.

^b^ ORs (95% CI) were derived from conditional logistic regression models stratified by matching identifiers (birth year, sex and recruitment center), and adjusted for birth weight, Townsend deprivation index, education level, and annual household income.

^c^ Individual height deviation (%): calculated by (rank of attained adult height-rank of genetically-determined height)/149610*100.

^d^ Height deficit: for an individual, rank of attained adult height < rank of genetically-determined height (yes or no).

Abbreviation: SD: standard deviation, CI: confidence interval, OR: Odd ratio.

**Table S5** Associations between asthma and attained adult height /individual height change (height deviation and deficit) stratified by diﬀerent characteristics

|  | Age at asthma diagnosis (years) | Attained adult height | Individual height deviation (%) ^a^ | Height deficit (yes or no) ^b^ |
| --- | --- | --- | --- | --- |
|  |  | β (95% CI)^c^ | β (95% CI)^c^ | OR (95% CI)^d^ |
| ***By birth year*** |  |  |  |  |
| 1936-1950 | Unexposed group | 0 (ref) | 0 (ref) | 1(ref) |
|  | ≤6 | -0.57 (-0.82~-0.32) | -2.30 (-3.40~-1.20) | 1.18 (1.07-1.30) |
|  | 7-12 | -0.36 (-0.63~-0.08) | -0.24 (-1.45~0.97) | 1.02 (0.92-1.14) |
|  | 13-18 | -0.25 (-0.63~0.14) | 0.30 (-1.39~1.98) | 1.01 (0.87-1.18) |
| 1951-1960 | Unexposed group | 0 (ref) | 0 (ref) | 1(ref) |
|  | ≤6 | -0.61 (-0.91~-0.31) | -2.09 (-3.37~-0.82) | 1.10 (0.97-1.24) |
|  | 7-12 | -0.46 (-0.75~-0.17) | -1.82 (-3.07~-0.58) | 1.12 (1.00-1.26) |
|  | 13-18 | -0.60 (-0.99~-0.21) | -0.58 (-2.26~1.11) | 0.96 (0.82-1.13) |
| 1961-1970 | Unexposed group | 0 (ref) | 0 (ref) | 1(ref) |
|  | ≤6 | -0.93 (-1.31~-0.55) | -1.36 (-3.00~0.28) | 1.02 (0.88-1.19) |
|  | 7-12 | -0.02 (-0.35~0.31) | 0.76 (-0.68~2.19) | 0.90 (0.79-1.03) |
|  | 13-18 | 0.01 (-0.37~0.39) | 0.07 (-1.57~1.71) | 0.97 (0.83-1.12) |
| ***By Townsend deprivation index*** | |  |  |  |
| Low | Unexposed group | 0 (ref) | 0 (ref) | 1(ref) |
|  | ≤6 | -0.41 (-0.70~-0.13) | -2.15 (-3.42~-0.89) | 1.15 (1.02-1.31) |
|  | 7-12 | -0.08 (-0.37~0.21) | 0.32 (-0.95~1.60) | 0.99 (0.87-1.12) |
|  | 13-18 | -0.29 (-0.67~0.09) | 0.42 (-1.24~2.08) | 0.96 (0.81-1.14) |
| Moderate | Unexposed group | 0 (ref) | 0 (ref) | 1(ref) |
|  | ≤6 | -0.92 (-1.21~-0.63) | -1.64 (-2.93~-0.36) | 1.14 (1.00-1.30) |
|  | 7-12 | -0.55 (-0.84~-0.25) | -0.62 (-1.91~0.66) | 1.05 (0.92-1.19) |
|  | 13-18 | -0.27 (-0.65~0.12) | -1.31 (-3.00~0.39) | 1.13 (0.94-1.35) |
| High | Unexposed group | 0 (ref) | 0 (ref) | 1(ref) |
|  | ≤6 | -0.67 (-0.97~-0.36) | -2.29 (-3.60~-0.98) | 1.10 (0.97-1.26) |
|  | 7-12 | -0.31 (-0.62~-0.01) | -1.33 (-2.63~-0.03) | 1.03 (0.90-1.17) |
|  | 13-18 | -0.25 (-0.64~0.14) | 0.66 (-1.00~2.32) | 0.96 (0.81-1.14) |
| ***By birth weight*** | |  |  |  |
| Low | Unexposed group | 0 (ref) | 0 (ref) | 1(ref) |
|  | ≤6 | -0.72 (-1.07~-0.37) | -2.08 (-3.6~-0.56) | 0.98 (0.82-1.17) |
|  | 7-12 | -0.39 (-0.76~-0.02) | -0.82 (-2.43~0.78) | 1.05 (0.87-1.25) |
|  | 13-18 | -0.31 (-0.75~0.12) | -0.61 (-2.50~1.27) | 1.14 (0.92-1.41) |
| Moderate | Unexposed group | 0 (ref) | 0 (ref) | 1(ref) |
|  | ≤6 | -0.81 (-1.2~-0.43) | -3.19 (-4.89~-1.48) | 1.03 (0.85-1.26) |
|  | 7-12 | -0.04 (-0.41~0.34) | 1.09 (-0.57~2.75) | 0.96 (0.79-1.16) |
|  | 13-18 | 0.03 (-0.45~0.51) | 0.57 (-1.56~2.69) | 0.80 (0.63-1.02) |
| High | Unexposed group | 0 (ref) | 0 (ref) | 1(ref) |
|  | ≤6 | -0.74 (-1.17~-0.32) | -2.10 (-3.92~-0.28) | 1.02 (0.81-1.28) |
|  | 7-12 | 0.09 (-0.32~0.51) | -0.79 (-2.57~0.99) | 0.97 (0.78-1.21) |
|  | 13-18 | -0.31 (-0.86~0.24) | -1.06 (-3.42~1.31) | 1.03 (0.76-1.39) |
| ***By annual household income, £*** | |  |  |  |
| <18 000 | Unexposed group | 0 (ref) | 0 (ref) | 1(ref) |
|  | ≤6 | -0.94 (-1.36~-0.52) | -2.94 (-4.74~-1.13) | 1.18 (0.96-1.46) |
|  | 7-12 | -0.51 (-0.96~-0.05) | -0.44 (-2.42~1.53) | 0.94 (0.75-1.18) |
|  | 13-18 | -0.38 (-0.95~0.20) | -0.93 (-3.39~1.52) | 1.07 (0.79-1.44) |
| 18 000- 52 000 | Unexposed group | 0 (ref) | 0 (ref) | 1(ref) |
|  | ≤6 | -0.70 (-0.96~-0.45) | -2.45 (-3.56~-1.35) | 1.13 (1.01-1.25) |
|  | 7-12 | -0.37 (-0.62~-0.11) | -1.46 (-2.58~-0.34) | 1.06 (0.95-1.17) |
|  | 13-18 | -0.22 (-0.55~0.12) | -1.07 (-2.51~0.37) | 0.98 (0.85-1.12) |
| ≥52 000 | Unexposed group | 0 (ref) | 0 (ref) | 1(ref) |
|  | ≤6 | -0.67 (-1.00~-0.34) | -1.92 (-3.37~-0.46) | 1.11 (0.96-1.30) |
|  | 7-12 | -0.07 (-0.36~0.23) | 0.70 (-0.60~1.99) | 0.94 (0.82-1.08) |
|  | 13-18 | -0.29 (-0.69~0.11) | 2.22 (0.44~4.00) | 0.91 (0.75-1.10) |

^a^ Individual height deviation (%): calculated by (rank of attained adult height-rank of genetically-determined height)/149610*100.

^b^ Height deficit: for an individual, rank of attained adult height < rank of genetically-determined height (yes or no).

^c^ β were estimate derived from linear mixed-effect models stratified by matching identifiers (birth year, sex and recruitment center), and adjusted for birth weight, Townsend deprivation index, education level, and annual household income.

^d^ ORs (95% CI) were derived from conditional logistic regression models stratified by matching identifiers (birth year, sex and recruitment center), and adjusted for birth weight, Townsend deprivation index, education level, and annual household income.

Abbreviation: CI: confidence interval, OR: Odd ratio.

**Table S6** Sensitivity analysis for the association between asthma and attained adult height / height change (height deviation and deficit) by removing unexposed individuals with asthma diagnosed after age of 18

|  | Full cohort (n=135095) | Male cohort (n=79043) | Female cohort (n=56052) |
| --- | --- | --- | --- |
|  |  |  |  |
| ***Outcome: Attained adult height (centimeter)*** | | | |
|  | β (95% CI)^a^ | β (95% CI)^a^ | β (95% CI)^a^ |
| Unexposed group | 0 (ref) | 0 (ref) | 0 (ref) |
| Exposed group, by age at asthma diagnosis (years) | |  |  |
| ≤ 2 | -1.03 (-1.39~-0.67) | -1.47 (-1.98~-0.95) | -0.55 (-1.05~-0.06) |
| 3-4 | -0.84 (-1.13~-0.55) | -1.01 (-1.42~-0.60) | -0.65 (-1.06~-0.25) |
| 5-6 | -0.43 (-0.68~-0.18) | -0.42 (-0.76~-0.08) | -0.46 (-0.85~-0.07) |
| 7-9 | -0.40 (-0.64~-0.16) | -0.30 (-0.61~0.01) | -0.56 (-0.93~-0.19) |
| 10-12 | -0.29 (-0.53~-0.05) | -0.16 (-0.47~0.14) | -0.55 (-0.93~-0.16) |
| 13-18 | -0.31 (-0.54~-0.09) | -0.08 (-0.40~0.24) | -0.57 (-0.87~-0.27) |
| ***Outcome: Individual height deviation (%)^b^*** | | |  |
|  | β (95% CI)^a^ | β (95% CI)^a^ | β (95% CI)^a^ |
| Unexposed group | 0 (ref) | 0 (ref) | 0 (ref) |
| Exposed group, by age at asthma diagnosis (years) | |  |  |
| ≤ 2 | -2.65 (-4.21~-1.08) | -4.63 (-7.06~-2.19) | -1.94 (-3.78~-0.10) |
| 3-4 | -2.89 (-4.15~-1.62) | -4.36 (-6.30~-2.42) | -2.88 (-4.38~-1.38) |
| 5-6 | -1.22 (-2.32~-0.11) | -2.06 (-3.66~-0.47) | -1.36 (-2.80~0.08) |
| 7-9 | -0.85 (-1.89~0.19) | -1.69 (-3.17~-0.21) | -1.15 (-2.52~0.22) |
| 10-12 | -0.39 (-1.43~0.65) | -0.88 (-2.34~0.59) | -0.51 (-1.93~0.90) |
| 13-18 | -0.13 (-1.10~0.84) | 0.69 (-0.84~2.22) | -1.40 (-2.51~-0.29) |
| ***Outcome: Height deficit (yes or no)^c^*** | | |  |
|  | OR (95% CI)^d^ | OR (95% CI)^d^ | OR (95% CI)^d^ |
| Unexposed group | 1 (ref) | 1 (ref) | 1 (ref) |
| Exposed group, by age at asthma diagnosis (years) | |  |  |
| ≤ 2 | 1.22 (1.05-1.41) | 1.40 (1.17-1.66) | 1.13 (0.95-1.36) |
| 3-4 | 1.17 (1.04-1.32) | 1.30 (1.13-1.49) | 1.22 (1.06-1.42) |
| 5-6 | 1.06 (0.96-1.17) | 1.18 (1.06-1.32) | 1.10 (0.95-1.26) |
| 7-9 | 1.03 (0.93-1.13) | 1.12 (1.01-1.24) | 1.09 (0.95-1.25) |
| 10-12 | 1.03 (0.93-1.13) | 1.10 (0.99-1.22) | 1.00 (0.87-1.15) |
| 13-18 | 0.98 (0.90-1.07) | 0.98 (0.88-1.10) | 1.07 (0.96-1.19) |

^a^. β were estimate derived from linear mixed-effect models stratified by matching identifiers (birth year, sex and recruitment center), and adjusted for birth weight, Townsend deprivation index, education level, and annual household income.

^b^. Individual height deviation (%): calculated by (rank of attained adult height-rank of genetically-determined height)/149610*100.

^c^. Height deficit: for an individual, rank of attained adult height < rank of genetically-determined height (yes or no).

^d^ ORs (95% CI) were derived from conditional logistic regression models stratified by matching identifiers (birth year, sex and recruitment center), and adjusted for birth weight, Townsend deprivation index, education level, and annual household income.

Abbreviation: CI: confidence interval, OR: Odd ratio.

**Table S7** Associations between asthma and height by use of inhaled glucocorticoids (ICS), after multiple imputation for the missing values of ICS use^a^

|  | ICS use | | No ICS use | |
| --- | --- | --- | --- | --- |
|  |  |  |  |  |
| ***Outcome: Attained adult height (centimeter)*** | | | | |
|  | **β (95% CI)^b^** | **average value^d^** | **β (95% CI)^b^** | **average value^d^** |
| Unexposed group | 0 (ref) | 0 (ref) | 0 (ref) | 0 (ref) |
| Exposed group, by age at asthma diagnosis (years) | | |  |  |
| ≤ 6 | -0.85 (-1.19~-0.51) | -0.81 (-1.13~-0.50) | -0.59 (-0.81~-0.37) | -0.59 (-0.79~-0.39) |
| 7-12 | -0.61 (-0.99~-0.23) | -0.52 (-0.86~-0.17) | -0.28 (-0.50~-0.07) | -0.23 (-0.43~-0.04) |
| 13-18 | -0.41 (-0.87~0.06) | -0.43 (-0.86~-0.003) | -0.17 (-0.45~0.11) | -0.22 (-0.47~0.04) |
| ***Outcome: Individual height deviation (%)^e^*** | | | | |
|  | **β (95% CI)^b^** |  | **β (95% CI)^b^** |  |
| Unexposed group | 0 (ref) | 1(ref) | 0 (ref) | 1(ref) |
| Exposed group, by age at asthma diagnosis (years) | | |  |  |
| ≤ 6 | -2.32 (-3.82~-0.82) | -2.42 (-3.79~-1.04) | -1.68 (-2.64~-0.72) | -1.86 (-2.75~-0.98) |
| 7-12 | -1.44 (-3.12~0.24) | -1.40 (-2.91~0.10) | -0.13 (-1.09~0.82) | -0.27 (-1.12~0.58) |
| *13-18* | -0.88 (-2.92~1.16) | -1.02 (-2.89~0.86) | 0.76 (-0.47~1.98) | 0.28 (-0.84~1.41) |
| ***Outcome: Height deficit (yes or no)^f^*** | | | | |
|  | **OR (95% CI)^c^** |  | **OR (95% CI)^c^** |  |
| Unexposed group | 1(ref) | 1(ref) | 1(ref) | 1(ref) |
| Exposed group, by age at asthma diagnosis (years) | | |  |  |
| ≤ 6 | 1.29 (1.12-1.48) | 1.20 (1.06~1.37) | 1.10 (1.01-1.21) | 1.08 (0.996~1.17) |
| 7-12 | 1.04 (0.89-1.21) | 1.04 (0.91~1.20) | 1.02 (0.93-1.11) | 1.02 (0.94~1.10) |
| 13-18 | 0.98 (0.81-1.19) | 0.97(0.81~1.15) | 0.98 (0.87-1.10) | 0.98 (0.89~1.09) |

^a^ We applied multiple imputation approach to deal with the missing ICS, based on the known characteristics of participants. Specifically, we replicated the imputation for 100 times, and obtained 100 datasets with imputed data for ICS use. Based on each imputed dataset, we re-run the subgroup analyses by ICS use (yes or no). Then, the results of imputed data were summarized through calculating the average values of the 100 estimated effect sizes and their 95% CIs.

^b^ β were estimate derived from Linear mixed-effect models stratified by matching identifiers (birth year, sex and recruitment center), and adjusted for birth weight, Townsend deprivation index, education level, and annual household income.

^c^ ORs (95% CI) were derived from conditional logistic regression models stratified by matching identifiers (birth year, sex and recruitment center), and adjusted for birth weight, Townsend deprivation index, education level, and annual household income.

^d^ Results of the imputation analyses were summarized through calculating the average values of the 100 estimated effect sizes and their 95% CIs.

^e^ Individual height deviation (%): calculated by (rank of attained adult height-rank of genetically-determined height)/149610*100.

^f^ Height deficit: for an individual, rank of attained adult height < rank of genetically-determined height (yes or no).

Abbreviation: SD: standard deviation, CI: confidence interval, OR: Odd ratio.

**Table S8** The associations between asthma and attained adult height / height change (height deviation and deficit), based on analysis of a full cohort design

|  | Full cohort (n=338117) | | | Men cohort (n=156599) | | | Women cohort (n=181518) | | |
| --- | --- | --- | --- | --- | --- | --- | --- | --- | --- |
|  |  |  |  |  |  |  |  |  |  |
| ***Outcome: Attained adult height (centimeter)*** | | | | | | | | | |
|  | **No. of individuals** | **Mean (SD)** | **β (95% CI)^a^** | **No. of individuals** | **Mean (SD)** | **β (95% CI)^a^** | **No. of individuals** | **Mean (SD)** | **β (95% CI)^a^** |
| Unexposed group | 324515 | 169 (9.24) | 0 (ref) | 148757^e^ | 176 (6.76) | 0 (ref) | 175758 | 163 (6.22) | 0 (ref) |
| Exposed group, by age at asthma diagnosis (years) | | | | | | | | | |
| ≤2 | 1150 | 169 (8.98) | -0.96 (-1.31~-0.60) | 599 | 175 (6.91) | -1.40 (-1.92~-0.89) | 551 | 162 (6.10) | -0.47 (-0.96~0.03) |
| 3-4 | 1783 | 169 (9.11) | -0.78 (-1.06~-0.49) | 950 | 175 (6.51) | -0.98 (-1.39~-0.57) | 833 | 162 (6.36) | -0.56 (-0.96~-0.16) |
| 5-6 | 2325 | 171 (9.28) | -0.38 (-0.63~-0.13) | 1420 | 176 (6.73) | -0.39 (-0.73~-0.06) | 905 | 163 (6.45) | -0.37 (-0.76~0.01) |
| 7-9 | 2650 | 171 (9.31) | -0.34 (-0.58~-0.11) | 1649 | 176 (6.64) | -0.28 (-0.59~0.04) | 1001 | 163 (6.49) | -0.48 (-0.84~-0.11) |
| 10-12 | 2621 | 172 (9.35) | -0.23 (-0.46~0.01) | 1684 | 177 (6.70) | -0.14 (-0.45~0.17) | 937 | 163 (6.40) | -0.44 (-0.82~-0.06) |
| 13-18 | 3073 | 170 (9.50) | -0.25 (-0.47~-0.03) | 1540 | 177 (6.90) | -0.05 (-0.37~0.28) | 1533 | 163 (6.30) | -0.47 (-0.77~-0.17) |
| ***Outcome: Individual height deviation (%)^c^*** | | | | | | |  | | |
|  | **No. of individuals** | **Mean (SD)** | **β (95% CI)^a^** | **No. of individuals** | **Mean (SD)** | **β (95% CI)^a^** | **No. of individuals** | **Mean (SD)** | **β (95% CI)^a^** |
| Unexposed group | 324515 | 0.23 (34.7) | 0 (ref) | 148757 | -0.06 (31.3) | 0 (ref) | 175758 | -0.02 (31.1) | 0 (ref) |
| Exposed group, by age at asthma diagnosis (years) | | | | | | | | | |
| ≤2 | 1150 | -0.77 (34.9) | -2.23 (-3.79~-0.68) | 599 | -3.54 (32.1) | -4.31 (-6.74~-1.87) | 551 | -1.59 (31.2) | -2.23 (-4.74~0.29) |
| 3-4 | 1783 | -1.12 (34.5) | -2.57 (-3.83~-1.32) | 950 | -2.81 (30.0) | -4.01 (-5.95~-2.08) | 833 | -2.69 (31.1) | -3.64 (-5.69~-1.59) |
| 5-6 | 2325 | -6.40 (34.3) | -0.98 (-2.08~0.12) | 1420 | 0.06 (30.9) | -1.73 (-3.31~-0.15) | 905 | -0.09 (32.0) | -1.54 (-3.51~0.42) |
| 7-9 | 2650 | -7.92 (34.0) | -0.70 (-1.73~0.33) | 1649 | 1.49 (31.5) | -1.39 (-2.86~0.09) | 1001 | 1.42 (31.6) | -1.31 (-3.18~0.56) |
| 10-12 | 2621 | -9.45 (34.3) | -0.20 (-1.24~0.83) | 1684 | 2.74 (31.2) | -0.57 (-2.03~0.89) | 937 | 2.12 (32.0) | -0.32 (-2.25~1.61) |
| 13-18 | 3073 | -3.78 (35.4) | 0.02 (-0.94~0.98) | 1540 | 3.97 (31.6) | 0.97 (-0.55~2.49) | 1533 | 1.67 (31.9) | -1.57 (-3.09~-0.06) |
| ***Outcome: Height deficit (yes or no)^d^*** | | | | | | | | | |
|  | **No. of individuals with** **height deficit/No. of total individuals (%)** | | **OR (95% CI)^b^** | **No. of individuals with** **height deficit/No. of total individuals (%)** | | **OR (95% CI)^b^** | **No. of individuals with** **height deficit/No. of total individuals (%)** | | **OR (95% CI)^b^** |
| Unexposed group | 164026/324515 (50.54) | | 1(ref) | 73953 (49.71) | | 1(ref) | 87389 (49.72) | | 1(ref) |
| Exposed group, by age at asthma diagnosis (years) | | | | | | | | | |
| ≤2 | 569/1150 (49.48) | | 1.19 (1.03-1.36) | 333/599 (55.59) | | 1.34 (1.14-1.58) | 287/551 (52.09) | | 1.14 (0.96-1.36) |
| 3-4 | 856/1783 (48.01) | | 1.15 (1.02-1.28) | 513/950 (54.00) | | 1.28 (1.13-1.46) | 446/833 (53.54) | | 1.24 (1.08-1.42) |
| 5-6 | 966/2325 (41.55) | | 1.03 (0.93-1.14) | 724/1420 (50.99) | | 1.17 (1.05-1.30) | 446/905 (49.28) | | 1.06 (0.93-1.22) |
| 7-9 | 1060/2650 (40.00) | | 1.02 (0.93-1.12) | 803/1649 (48.70) | | 1.13 (1.02-1.24) | 478/1001 (47.75) | | 1.07 (0.94-1.22) |
| 10-12 | 1021/2621 (38.95) | | 1.04 (0.94-1.14) | 798/1684 (47.39) | | 1.09 (0.99-1.21) | 433/937 (46.21) | | 0.99 (0.87-1.13) |
| 13-18 | 1391/3073 (45.27) | | 0.97 (0.89-1.06) | 701/1540 (45.52) | | 0.99 (0.90-1.10) | 701/1533 (45.73) | | 1.01 (0.91-1.12) |

^a^ β were estimate derived from linear regression models adjusted for birth year, sex, recruitment center, birth weight, Townsend deprivation index, education level, and annual household income.

^b^ ORs and 95% CI were derived from logistic regression models adjusted for birth year, sex, recruitment center, birth weight, Townsend deprivation index, education level, and annual household income.

^c^ Individual height deviation (%): calculated by (rank of attained adult height-rank of genetically-determined height)/338117*100.

^d^ Height deficit: for an individual, rank of attained adult height < rank of genetically-determined height (yes or no).

Abbreviation: SD: standard deviation, CI: confidence interval, OR: Odd ratio.
